# Extracellular Fluid/Intracellular Fluid Volume Ratio as a Novel Risk Indicator for All-Cause Mortality and Cardiovascular Disease in Hemodialysis Patients

**DOI:** 10.1371/journal.pone.0170272

**Published:** 2017-01-18

**Authors:** Eun-Jung Kim, Myung-Jin Choi, Jeoung-Hwan Lee, Ji-Eun Oh, Jang-Won Seo, Young-Ki Lee, Jong-Woo Yoon, Hyung-Jik Kim, Jung-Woo Noh, Ja-Ryong Koo

**Affiliations:** 1 Department of Internal Medicine, Hallym Kidney Research Institute, College of Medicine, Hallym University, Chuncheon, Korea; 2 Division of Nephrology, Dongtan Sacred Heart Hospital, Hallym University Medical Center, Hwaseong-si, Korea; The University of Tokyo, JAPAN

## Abstract

**Background:**

In hemodialysis patients, fluid overload and malnutrition are accompanied by extracellular fluid (ECF) expansion and intracellular fluid (ICF) depletion, respectively. We investigated the relationship between ECF/ICF ratio (as an integrated marker reflecting both fluid overload and malnutrition) and survival and cardiovascular disease (CVD) in the context of malnutrition-inflammation-arteriosclerosis (MIA) complex.

**Methods:**

Seventy-seven patients from a single hemodialysis unit were prospectively enrolled. The ECF/ICF volume was measured by segmental multi-frequency bioimpedance analysis. MIA and volume status were measured by serum albumin, C-reactive protein (CRP), pulse wave velocity (PWV) and plasma B-type natriuretic peptide (BNP), respectively.

**Results:**

The mean ECF/ICF ratio was 0.56±0.06 and the cut-off value for maximum discrimination of survival was 0.57. Compared with the low ECF/ICF group, the high ECF/ICF group (ratio≥0.57, 42%) had higher all-cause mortality, CVD, CRP, PWV, and BNP, but lower serum albumin. During the 5-year follow-up, 24 all-cause mortality and 38 CVD occurred (18 and 24, respectively, in the high ECF/ICF group versus 6 and 14 respectively in the low ECF/ICF group, P<0.001). In the adjusted Cox analysis, the ECF/ICF ratio nullifies the effects of the MIA and volume status on survival and CVD and was an independent predictor of all-cause mortality and CVD: hazard ratio (95% confidence interval); 1.12 (1.01–1.25) and 1.09 (1.01–1.18) for a 0.01 increase in the ECF/ICF ratio. The degree of malnutrition (albumin), inflammation (CRP), arteriosclerosis (PWV), and fluid overload (BNP) were correlated well with the ECF/ICF ratio.

**Conclusions:**

Hemodialysis patients with high ECF/ICF ratio are not only fluid overloaded, but malnourished and have stiff artery with more inflammation. The ECF/ICF ratio is highly related to the MIA complex, and is a major risk indicator for all-cause mortality and CVD.

## Introduction

For a long time, fluid overload and malnutrition have been known to be major risk factors for morbidity and mortality in chronic hemodialysis patients [1–3]. Recently, a strong association between malnutrition, inflammation, and arteriosclerosis/atherosclerosis (the so-called MIA syndrome) have been described and proposed as the main causes of morbidity and mortality in chronic hemodialysis patients [1, 4–6]. Among the MIA components, inflammation seems to play a pivotal role in the pathogenesis of malnutrition and arteriosclerosis by the following mechanisms: (1) inflammatory response is responsible for malnutrition by increased protein catabolism and muscle wasting [4, 6]; and (2) uremic inflammation is known to promote extra-osseous deposition of calcium to vessel walls, resulting in vascular calcification and arteriosclerosis [7].

There are also several supporting data showing the causal relationships between extracellular fluid (ECF) overload and the MIA complex [8–10]. It has been proposed that fluid overload act as an inflammatory stimulus by immune activation resulting from poor tissue perfusion, and bowel edema- induced translocation of bowel endotoxins into the circulation [9]. Fluid overload might also play an important role in the development of arteriosclerosis, through the increase in vessel wall stress caused by arterial distension (Laplace’s law) [10]. Conversely, hypoalbuminemia and increased vascular permeability caused by inflammation will enhance extravascular fluid shift, resulting in ECF volume overload [11]. In addition, malnutrition caused by inflammation could deplete body cell mass, which eventually leads to the decrease in intracellular fluid (ICF) volume, and the relative increase in ECF/ICF volume ratio [12, 13].

It is possible to distinguish between total body fluid (TBF) and ECF with multi-frequency bioimpedance analysis (MF-BIA), by using the resistance of cell membranes to relatively low-frequency currents. At high frequencies, currents flow across both intra- and extracellular spaces; however, at low frequencies, currents flow mainly through extracellular space; allowing the assessment of ECF, ICF, and TBF [14].

**Fig 1:**
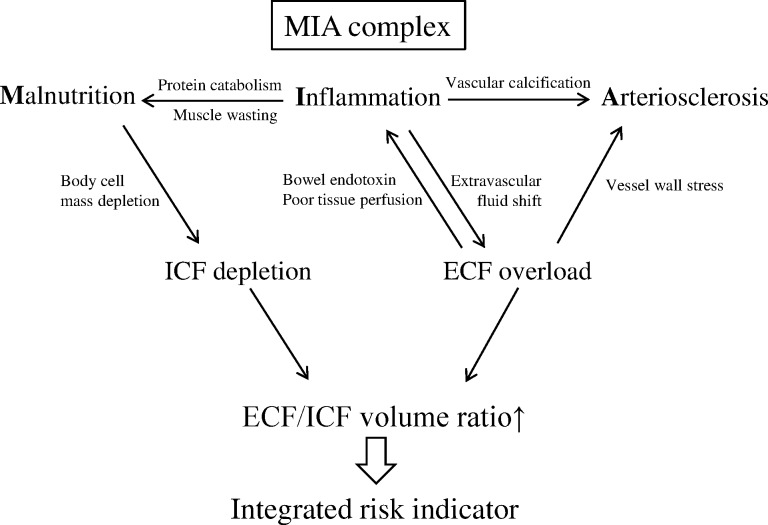
The possible relationship between the MIA complex and ECF/ICF volumes. As an integrated marker of fluid volume overload and malnutrition, the ECF/ICF ratio might be highly-related to the MIA complex, and can be a major risk indicator of morbidity and mortality in chronic hemodialysis patients.

In view of the above considerations, the ECF/ICF ratio measured by MF-BIA may be highly related to the MIA complex, and could be defined as a novel integrated marker reflecting both fluid overload and malnutrition (Fig 1). In this study, we investigated the relationship of ECF/ICF ratio to survival and cardiovascular disease (CVD) in the context of MIA complex in chronic hemodialysis patients.

## Materials and Methods

### Patients

This study was a single center, prospective, longitudinal study with patients recruited from the hemodialysis unit of Hallym University Hospital (Chuncheon, Korea). The protocol of this study was approved by the Institutional Review Board of Hallym University Hospital (Chuncheon, Korea) and all patients provided written informed consent. Patients were eligible for inclusion if they (1) had been on hemodialysis for at least 6 months, (2) had no clinical CVD for 3 months preceding enrollment, (3) were 18 years of age or older, and (4) agreed to participate in the follow-up study. Patients who underwent renal transplantation and patients who moved were censored on the day of transplantation or departure. Among the 86 eligible hemodialysis patients, 77 patients were prospectively enrolled in the study from January 2007 and followed up for 5 years. Nine patients were excluded based on the exclusion criteria, namely patients with an underlying malignancy, Child’s C liver cirrhosis or patients with incomplete data. During the follow-up period, 8 patients were censored due to renal transplantation (n = 4) and transfer to other hemodialysis unit (n = 4). The causes of chronic renal failure were diabetic nephropathy in 29 patients (37.7%), chronic glomerulonephritis in 25 patients (32.5%), hypertension in 17 patients (22.1%), and polycystic kidney disease in 1 patient (1.3%). The remainder was attributed to unknown and/or miscellaneous causes. All participating patients underwent three 4-hour sessions of hemodialysis per week using bicarbonate-buffered dialysate. The dialysis membrane used for all patients was composed of polysulfone (Fresenius F6HPS; Fresenius, Bad Homburg, Germany), with a surface area of 1.3 m^2^. All the biochemical measurements and assessments of fluid volume, MIA component and cardiovascular function were performed at mid-week (Wednesday or Thursday) after a short (1 day) inter-dialytic interval.

### Assessments of fluid volume and MIA components

The ECF, ICF, and TBF volumes were measured by segmental MF-BIA, which was performed 30 minutes before hemodialysis with the subject in the standing position. Plasma B-type natriuretic peptide (BNP) was also measured to evaluate the fluid volume status.

Among the MIA components, the severity of malnutrition (the first MIA component) was assessed by the mean value of pre-dialysis serum albumin levels, which were measured three times at weekly intervals. The modified subjective global assessment (SGA) used in the Canada-USA Peritoneal Dialysis Study [15] was also used to evaluate the overall protein-energy nutritional status. SGA includes four items (weight loss over the past 6 months, anorexia, subcutaneous fat, and muscle mass) scored on a seven-point Likert scale. Scores of 1 to 2 represent severe malnutrition; 3 to 5, moderate to mild malnutrition; and 6 to 7, normal nutrition. The normalized protein catabolic rate (nPCR) was calculated as a marker of protein intake, by using a variable-volume single-pool urea kinetic calculator on a web site (Hypertension Dialysis Clinical Nephrology; http://www.hdcn.com/).

The degree of an inflammatory reaction (the second MIA component) was assessed by a mean value of serum C-reactive protein (CRP) levels, which were also measured three times at weekly intervals.

Pre-dialysis brachial-ankle pulse wave velocity (PWV) was measured as an indicator of arteriosclerosis (the third MIA component), using a volume plethysmographic apparatus (Form/ABI, Colin, Co. Ltd., Komaki, Japan). Details of the methodology were described elsewhere [16]. Each subject was examined while resting in the supine position. Cuffs were wrapped on both the brachia and ankles. Pulse volume waveforms at the brachium and ankle were recorded using a semiconductor pressure sensor. Brachial-ankle PWV was measured after at least 5 minutes of rest.

### Segmental MF-BIA

In this study, we used segmental MF-BIA, which measured the resistance of the trunk or each limb separately. It is a more appropriate approach to monitor body water during hemodialysis than whole-body BIA, as changes in local resistance can be allocated to segments with uniformed geometry and resistivity [17]. Water volumes were calculated by means of a population-based regression equation using the impedance index (height squared/resistance).

Eight stainless steel tactile electrodes were used to measure the segmental impedance of the trunk and extremity using Inbody2.0 (Biospace Co, Seoul, Korea), as described in our previous study [18]. Two electrodes were in contact with the palm and thumb of each hand, while another two with the anterior and posterior aspects of the sole of each foot. Impedance was measured at frequencies of 5, 50, 250, and 500 kHz in the standing position. The validity of the method has been reported using sodium bromide and deuterium oxide dilutions [14].

To decrease operator variability, all MF-BIA tests were performed by the same operator, trained in MF-BIA study. Measurements were taken using same equipment, room, and temperature, in the same position.

### Echocardiography, ankle-brachial index and biochemical analysis

Echocardiography was performed 30 min prior to hemodialysis by a single experienced cardiologist who was blinded to the patients’ clinical and laboratory data. The ejection fraction and left ventricular mass index (LVMI) were measured according to the recommendations of the American Society of Echocardiography [19].

As a surrogate marker of atherosclerotic peripheral arterial disease, pre-dialytic ankle brachial index (ABI) was measured using Omron VP-1000 Vascular Profilter (Colin, Co. Ltd., Komaki, Japan).

Blood samples for biochemical analysis were obtained from the patient’s arteriovenous access or tunneled dialysis catheter before hemodialysis. Serum CRP level was determined using a particle enhanced immunoturbidimetric assay (Roche diagnostics, USA). Plasma B-type natriuretic peptide (BNP) concentration was determined using the ADVIA Centaur^®^ BNP immunoassay on the ADVIA Centaur analyzer (Siemens Healthcare Diagnostics Inc., Deerfield, IL, USA). Plasma intact-parathyroid hormone (PTH) was measured using an immunochemiluminometric assay on the ADVIA Centaur analyzer (Siemens Healthcare Diagnostics Inc., Deerfield, IL, USA). Urea, albumin, hemoglobin, calcium, phosphate and total CO_2_ were measured by standard techniques. Each measurement was repeated three times at weekly intervals; the mean value derived was then used for statistical analysis.

### Outcomes

The primary endpoint was time to all-cause mortality. The secondary endpoint was time to fatal and non-fatal cardiovascular events requiring admission. Cardiovascular events were defined as ischemic heart disease, congestive heart failure, stroke, and peripheral vascular disease. The diagnosis of ischemic heart disease was based on the clinical history, electrocardiograms, stress test, cardiac biomarker (troponin I), and coronary angiography. The diagnosis of congestive heart failure was determined by the clinical history and echocardiography. Stroke was identified by clinical signs of neurological dysfunction and abnormalities in brain imaging studies. Symptomatic peripheral vascular disease was determined from ABI, CT, and percutaneous angiography. Hemodialysis vascular access dysfunction was not included as an outcome measure.

### Statistical analysis

Data analysis was performed using the SPSS 17.0 software package (SSPS Inc., Chicago, IL, USA). Data measured only at enrollment were used for analysis; although, biochemical measurement was repeated three times at weekly intervals and the mean value was used for statistical analysis to minimize the inter-dialytic variability, it also represents data at enrollment (during 1^st^ 3 weeks) and no follow-up variable was included in the analysis.

All data are expressed as mean ± SD and a P value of <0.05 was considered statistically significant. The Kolmogorov-Smirnov test was used to verify the normality of the distribution of continuous variables. Variables that did not show normal distribution were serum CRP, plasma BNP and intact PTH levels, which were expressed as median and interquartile range (IQR). Nonparametric comparison and correlation tests were used for these variables. The differences between the high and low ECF/ICF ratio groups were assessed using unpaired Student’s *t*-test, Mann–Whitney U test, and chi-square test. The correlations of the ECF/ICF ratio with MIA components and BNP were assessed using Pearson’s and Spearman’s correlation coefficients. The difference in survival according to the ECF/ICF ratio was estimated using the Kaplan-Meier product-limit method and compared using the Mantel (log-rank) test. The prognostic factors of survival were identified using the univariate and multivariate Cox proportional hazards regression model. Estimated hazard ratios and their 90% confidence intervals were calculated with the estimated regression coefficients and standard errors. For maximum discrimination of survival by ECF/ICF ratio, an ECF/ICF ratio cut-off value was calculated by the maximally selected rank statistics method, available in the R maxstat package (R version 3.3.1, The R foundation for Statistical Computing).

## Results

The characteristics of the cohort at the time of inclusion are shown in Table 1. The mean ECF/ICF ratio was 0.56±0.06. During the 5-year follow-up, 24 all-cause mortality and 38 new cardiovascular events occurred. The cut-off ECF/ICF ratio for maximum discrimination of all-cause mortality by survival analysis was 0.57. The comparisons according to the cut-off ECF/ICF ratio are shown in Table 2. Compared with the low ECF/ICF group (ECF/ICF ratio<0.57), the high ECF/ICF group (ECF/ICF ratio≥0.57) had higher all-cause mortality, CVD, systolic blood pressure (BP), pulse pressure, number of antihypertensive pills, and LVMI, but lower hemodialysis duration, hemoglobin, BUN, serum creatinine, plasma intact PTH, and serum phosphate. The mean age and proportions of patients with diabetes and prior CVD were also higher in the high ECF/ICF ratio group.

**Table 1:** Baseline characteristics of the study population.

Variables	Mean±SD, % (n) or median (IQR)	Variables	Mean±SD, % (n)or median (IQR)
Age (years)	52.6±12.5	ECF/ICF	0.56±0.06
Women (%, n)	48.1 (37)	ECF/dry weight	0.21±0.02
Diabetes (%, n)	37.7 (29)	TBF/dry weight	0.58±0.06
Duration of dialysis (month)	51.1±36.9	BUN (mg/dL)	59.4±13.7
Prior CVD (%, n)	28.6 (22)	Serum creatinine (mg/dL)	8.23±1.99
LV mass index (g/m^2^)	124.5±39.5	Plasma total CO_2_ (mmol/L)	17.6±1.9
LV ejection fraction (%)	69.3±8.9	Serum calcium (mg/dL)	8.71±0.71
Pre-dialytic SBP (mmHg)	155.9±24.0	Serum phosphate (mg/dL)	4.13±1.35
Pre-dialytic DBP (mmHg)	85.7±10.1	Serum CRP (mg/L, median)	0.70 (0.06–2.45)
Ankle brachial index	1.05±0.14	Serum albumin (g/dL)	3.87±0.34
SGA score	4.90±1.36	nPCR	1.07±0.17
Kt/V	1.60±0.25	Plasma BNP (pg/mL, median)	441 (109–711)
Hemoglobin (g/dL)	9.6±0.8	Pulse pressure (mmHg)	70.2±20.0
Plasma iPTH (pg/mL, median)	81 (37–175)	Pulse wave velocity (cm/sec)	1894±582

Note: Data are presented as the mean±SD, % (n) or median (IQR, interquartile range). Conversion factors for units: creatinine in mg/dL to μmol/L, ⅹ88.4; urea nitrogen in mg/dL to μmol/L, ⅹ0.357; calcium in mg/dL to mmol/L, ⅹ0.249; phosphorus in mg/dL to mmol/L, ⅹ0.3229.

Abbreviations: BNP, brain natriuretic peptide; SBP, systolic blood pressure; DBP, diastolic blood pressure; CRP, C-reactive protein; CVD, cardiovascular disease; ECF/ICF, extracellular fluid/intracellular fluid; LV, left ventricular; nPCR, normalized protein catabolic rate; iPTH, intact parathyroid hormone; PWV, pulse wave velocity; SGA, subjective global assessment; BUN, blood urea nitrogen; TBF, total body fluid.

**Table 2:** Comparisons according to the cut-off ECF/ICF ratio.

Variables	ECF/ICF<0.57	ECF/ICF≥0.57	P
Number of patients (%, n)	58 (45)	42 (32)	
All-cause mortality (%, n)	13.3 (6)	56.3 (18)	<0.001
Cardiac disease	4.4 (2)	12.5 (4)	
Stroke	4.4 (2)	15.6 (5)	
Infection	2.2 (1)	21.9 (7)	
Undefined	2.2 (1)	6.3 (2)	
New CVD (%, n)	31.1 (14)	75.0 (24)	<0.001
Ischemic heart disease	11.1 (5)	21.9 (7)	
Congestive heart failure	6.7 (3)	28.1 (9)	
Stroke	6.7 (3)	21.9 (7)	
Peripheral vascular disease	6.7 (3)	3.1 (1)	
Age (years)	49.3±11.5	57.3±12.5	0.005
Women (%, n)	40.0 (18)	59.4 (19)	0.11
Diabetes mellitus (%, n)	13.3 (6)	71.9 (23)	<0.001
Prior CVD (%, n)	8.9 (4)	56.3 (18)	<0.001
Duration of dialysis (month)	57.6±39.8	40.0±29.0	0.04
Kt/V	1.58±0.25	1.64±0.26	0.35
Hemoglobin (g/dL)	9.78±0.89	9.30±0.56	0.008
BUN (mg/dL)	63.2±12.0	53.2±12.6	0.002
Serum creatinine (mg/dL)	8.93±1.98	7.06±1.37	<0.001
Plasma total CO_2_ (mmol/L)	17.1±1.7	18.10±1.9	0.02
Plasma intact PTH (pg/mL)	170.0±139.2	53.6±72.1	<0.001*
Serum calcium (mg/dL)	8.68±0.76	8.77±0.64	0.64
Serum phosphate (mg/dL)	4.42±1.45	3.64±1.01	0.03
Ankle brachial index	1.05±0.10	1.05±0.19	0.95
Pre-dialytic SBP (mmHg)	146.9±21.5	168.6±21.7	<0.001
Pre-dialytic DBP (mmHg)	85.8±9.0	85.6±11.6	0.95
Pulse pressure (mmHg)	61.1±16.1	83.0±18.1	<0.001
Number of antihypertensive pills	2.25±2.48	4.11±2.50	0.003
LV ejection fraction (%)	69.9±7.7	68.4±10.5	0.51
LV mass index	109.6±37.2	149.3±30.3	<0.001

Note: Data are presented as the mean±SD or as % (n).

*analyzed by Mann–Whitney U test.

Conversion factors for units: creatinine in mg/dL to μmol/L, ⅹ88.4; urea nitrogen in mg/dL to μmol/L, ⅹ0.357; calcium in mg/dL to mmol/L, ⅹ0.249; phosphorus in mg/dL to mmol/L, ⅹ0.3229.

Abbreviations: BNP, brain natriuretic peptide; SBP, systolic blood pressure; DBP, diastolic blood pressure; CRP, C-reactive protein; ECF/ICF, extracellular fluid/intracellular fluid; LV, left ventricular; nPCR, normalized protein catabolic rate; PTH, parathyroid hormone; PWV, pulse wave velocity; SGA, subjective global assessment; BUN, blood urea nitrogen.

Among the MIA and fluid volume components, serum albumin, SGA score, and nPCR were significantly lower, while serum CRP, PWV, and plasma BNP were significantly higher in the high ECF/ICF ratio group compared with the low ECF/ICF ratio group (Table 3). In correlation analysis between the various fluid volume ratios (ECF/ICF, ECF/dry weight, TBF/dry weight) and the individual MIA components and plasma BNP (Table 4), the ECF/ICF ratio was well correlated with all MIA components and plasma BNP. However the dry weight adjusted sole ECF volume (ECF/dry weight) was correlated only with the SGA score, nPCR and plasma BNP, while the dry weight adjusted sole TBF volume (TBF/dry weight) was correlated with none of the MIA components and plasma BNP.

**Table 3:** Comparisons of the MIA components and fluid volume statusaccording to the cut-off ECF/ICF ratio.

Variables	ECF/ICF<0.57	ECF/ICF≥0.57	P
MIA components			
Serum albumin (g/dL)	3.98±0.26	3.70±0.37	<0.001
SGA score	5.58±1.03	3.94±1.19	<0.001
nPCR	1.12±0.16	1.00±0.15	0.003
Serum CRP (mg/L)	0.95±1.41	2.90±3.15	<0.001*
PWV (m/sec)	1647±404	2288±699	<0.001
Fluid volume status			
Plasma BNP (pg/mL)	372±469	1160±1220	<0.001*

Note: Data are presented as the mean±SD.

*analyzed by Mann–Whitney U test.

Abbreviations: BNP, brain natriuretic peptide; CRP, C-reactive protein; ECF/ICF, extracellular fluid/intracellular fluid; MIA, malnutrition-inflammation-arteriosclerosis; nPCR, normalized protein catabolic rate; PWV, pulse wave velocity; SGA, subjective global assessment.

**Table 4:** Correlations (r) between the various fluid volume ratios and the individual MIA components and plasma BNP.

Fluid volume ratios	MIA components					Plasma BNP
	Serum albumin	SGA score	nPCR	Serum CRP	PWV	
ECF/ICF	-0.547^a^	-0.674^a^	-0.446^a^	0.537^a^*	0.690^a^	0.642^a^*
ECF/Dry weight	-0.061	-0.311^c^	-0.309^c^	0.127*	0.166	0.419^b^*
TBF/Dry weight	0.176	-0.015	-0.188	-0.162*	-0.151	0.091*

^a^P<0.001.

^b^P<0.01.

^c^P<0.05.

*analyzed by Spearman’s correlation test.

Abbreviations: BNP, brain natriuretic peptide; CRP, C-reactive protein; ECF/ICF, extracellular fluid/intracellular fluid; MIA, malnutrition-inflammation-arteriosclerosis; nPCR, normalized protein catabolic rate; PWV, pulse wave velocity; SGA, subjective global assessment; TBF, total body fluid.

**Fig 2:**
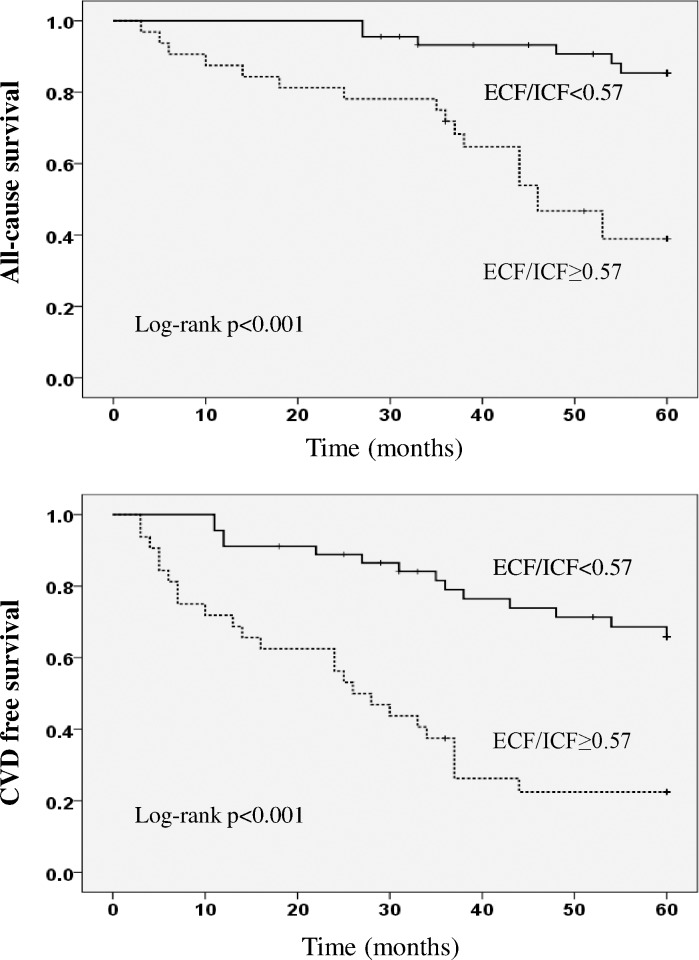
All-cause and CVD free survival according to the ECF/ICF ratio. A higher ECF/ICF ratio (≥0.57) was associated with a significantly lower all-cause and CVD free survival (log rank P<0.001). CVD, cardiovascular disease; ECF/ICF, extracellular fluid/intracellular fluid.

Fig 2 shows the Kaplan-Meier curves of all-cause and CVD free survival in the study population according to the cut-off ECF/ICF ratio. A higher ECF/ICF ratio was associated with a significantly lower all-cause and CVD free survival (all-cause mortality and new CVD were 18 and 24, respectively, in the high ECF/ICF ratio group, versus 6 and 14, respectively, in the low ECF/ICF ratio group; log rank P<0.001).

The results of the unadjusted and adjusted Cox proportional hazard analysis of all-cause mortality and new CVD are listed in Tables 5 and 6. Cox model 1 (Table 5) is composed of the 3 MIA components (i.e., serum albumin, CRP, and PWV), fluid volume marker (plasma BNP), and ECF/ICF ratio. Cox model 2 (Table 6) is composed of the ECF/ICF ratio and other major cardiovascular risk factors in chronic hemodialysis patients.

**Table 5:** Unadjusted univariate and adjusted multivariate Cox proportional hazard model 1 for all-cause mortality and new CVD; Model 1 is composed of the 3 MIA components, fluid volume marker (plasma BNP), and ECF/ICF ratio.

Model 1	All-cause mortality			New CVD	
Risk factors	Hazard Ratio (95% CI)		P	Hazard Ratio (95% CI)	P
Unadjusted univariate					
Malnutrition (serum albumin, /0.1g/dL)		0.72 (0.64–0.81)	<0.001	0.70 (0.62–0.80)	<0.001
Inflammation (serum CRP, /1mg/L)		1.36 (1.20–1.53)	<0.001	1.47 (1.31–1.66)	<0.001
Arteriosclerosis (PWV, /100m/sec)		1.14 (1.07–1.20)	<0.001	1.18 (1.11–1.25)	<0.001
Volume (Plasma BNP, /100pg/mL)		1.04 (1.01–1.08)	0.01	1.03 (1.00–1.06)	0.09
ECF/ICF (/0.01)		1.18 (1.11–1.25)	<0.001	1.17 (1.11–1.24)	<0.001
Adjusted multivariate					
Malnutrition (serum albumin, /0.1g/dL)		0.80 (0.63–1.01)	0.06	0.88 (0.73–1.06)	0.16
Inflammation (serum CRP, /1mg/L)		1.24 (0.96–1.60)	0.11	1.20 (0.97–1.49)	0.10
Arteriosclerosis (PWV, /100m/sec)		0.98 (0.86–1.11)	0.71	1.08 (0.97–1.20)	0.19
Volume (plasma BNP, /100pg/mL)		1.03 (0.93–1.13)	0.61	0.99 (0.92–1.07)	0.76
ECF/ICF (/0.01)		1.14 (1.03–1.26)	0.01	1.11 (1.02–1.19)	0.01

Abbreviations: BNP, brain natriuretic peptide; CRP, C-reactive protein; CVD, cardiovascular disease; ECF/ICF, extracellular fluid/intracellular fluid; MIA, malnutrition-inflammation-arteriosclerosis; PWV, pulse wave velocity.

**Table 6:** Unadjusted univariate and adjusted multivariate Cox proportional hazard model 2 for all-cause mortality and new CVD. In model 2, with other major cardiovascular risk factors, the ECF/ICF ratio (instead of individual MIA components and plasma BNP) was included as an integrated marker reflecting both the MIA complex and fluid volume status.

Model 2		All-cause mortality				New CVD		
Risk factors	Hazard Ratio (95% CI)			P	Hazard Ratio (95% CI)			P
Unadjusted univariate								
ECF/ICF (/0.01)		1.18 (1.11–1.25)	<0.001			1.17 (1.11–1.24)	<0.001	
ECF/ICF≥0.57		6.11 (2.41–15.49)	<0.001			4.02 (2.06–7.84)	<0.001	
ECF/dry weight (/0.01 L/kg)		1.08 (0.86–1.36)	0.52			1.07 (0.90–1.27)	0.48	
TBF/dry weight (/0.01 L/kg)		9.31 (0.86–1.01)	0.09			0.94 (0.88–1.00)	0.05	
Diabetes mellitus		4.15 (1.77–9.71)	0.001			4.88 (2.49–9.57)	<0.001	
Prior CVD		8.36 (3.48–20.10)	<0.001			6.77 (3.44–13.34)	0.001	
Plasma total CO_2_ (/1 meq/L)		1.01 (0.81–1.27)	0.93			1.15 (0.96–1.38)	0.14	
Age (/10 year)		2.00 (1.37–2.90)	<0.001			1.91 (1.42–2.56)	<0.001	
Women		2.33 (0.99–5.47)	0.05			1.52 (0.80–2.90)	0.20	
Hemodialysis duration (/1 month)		1.00(0.98–1.01)	0.73			1.00 (0.99–1.01)	0.57	
Pre-dialytic SBP (/10 mmHg)		1.33 (1.12–1.58)	0.001			1.32 (1.15–1.51)	<0.001	
Pre-dialytic DBP (/10 mmHg)		0.90 (0.60–1.37)	0.90			1.00 (0.73–1.38)	0.99	
Ankle brachial index (/0.1)		0.69 (0.52–0.92)	0.01			0.72 (0.57–0.90)	0.004	
LV ejection fraction (/10%)		0.98 (0.57–1.71)	0.95			0.70 (0.46–1.06)	0.09	
LV mass index (/10 g/m^2^)		1.00 (0.88–1.12)	0.96			1.03 (0.95–1.13)	0.44	
Kt/V (/0.1)		1.07 (0.88–1.31)	0.50			0.98 (0.85–1.12)	0.98	
Plasma intact PTH (/10 pg/mL)		0.97 (0.93–1.02)	0.27			0.97 (0.94–1.00)	0.07	
Serum calcium X phosphate (/1)		0.99 (0.95–1.03)	0.57			1.00 (0.97–1.03)	0.87	
Serum calcium (/1 mg/dL)		1.09 (0.55–2.16)	0.81			1.08 (0.65–1.80)	0.77	
Serum phosphate (/1 mg/dL)		0.95 (0.64–1.40)	0.78			1.02 (0.78–1.35)	0.87	
Adjusted multivariate								
ECF/ICF (/0.01)		1.12 (1.01–1.25)	0.03			1.09 (1.01–1.18)	0.03	
Diabetes mellitus		0.48 (0.11–2.10)	0.33			0.80 (0.26–2.44)	0.69	
Prior CVD		7.59 (2.28–25.32)	0.001			2.73 (1.10–6.75)	0.03	
Age (/10 year)		1.12 (0.67–1.89)	0.66			1.38 (0.94–2.03)	0.10	
Pre-dialytic SBP (/10 mmHg)		1.23 (0.90–1.67)	0.20			1.20 (0.98–1.47)	0.09	
Ankle brachial index (/0.1)		0.78 (0.59–1.04)	0.09			0.93 (0.74–1.16)	0.49	

Abbreviations: CVD, cardiovascular disease; ECF/ICF, extracellular fluid/intracellular fluid; LV, left ventricular; MIA, malnutrition-inflammation-arteriosclerosis; PTH, parathyroid hormone; SBP, systolic blood pressure; DBP, diastolic blood pressure; TBF, total body fluid.

In the unadjusted univariate Cox model 1, the ECF/ICF ratio, MIA components, and plasma BNP were independent predictors of all-cause mortality. ECF/ICF ratio and MIA components were also independent predictors of new CVD. In the adjusted multivariate Cox model 1, the ECF/ICF ratio is a sole risk factor nullifying the effects of the all 3 MIA components and plasma BNP on all-cause mortality and new CVD.

As the ECF/ICF ratio nullified the effects of the MIA components and plasma BNP on all-cause mortality and new CVD in the adjusted multivariate Cox model 1, the ECF/ICF ratio (instead of individual MIA components and plasma BNP) was included as an integrated marker reflecting both the MIA complex and fluid volume status in Cox model 2. In the unadjusted univariate Cox model 2, the ECF/ICF ratio, diabetes, prior CVD, age, systolic BP, and low ankle brachial index were independent predictors of all-cause mortality and new CVD. In the adjusted multivariate Cox model 2 (composed of variables with a P less than 0.05 in the unadjusted univariate Cox model 2), only the ECF/ICF ratio and prior CVD were independent predictors of all-cause mortality and new CVD.

Re-analysis of the adjusted multivariate Cox model 1 by replacing ECF/ICF ratio by ECF/TBF ratio to compare the 2 predictor showed that, like the ECF/ICF ratio, the ECF/TBF ratio is a sole risk factor nullifying the effects of the all 3 MIA components and plasma BNP on all-cause mortality and new CVD: hazard ratio (95% confidence interval); 1.43 (1.07–1.90) and 1.30 (1.05–1.61) for a 0.01 increase in the ECF/TBF ratio. However, contrary to the ECF/ICF ratio, the ECF/TBF ratio was not an independent predictor of all-cause mortality and new CVD in the adjusted multivariate Cox model 2: hazard ratio (95% confidence interval); 1.19 (0.83–1.73) and 1.08 (0.84–1.39) for a 0.01 increase in the ECF/TBF ratio.

## Discussion

This was the first study to show that the ECF/ICF ratio, as measured by MF-BIA, can be used as a novel risk indicator for all-cause mortality and CVD in chronic hemodialysis patients. In this population of patients, higher ECF volume represents fluid excess, while lower ICF volume represents malnutrition (or low body cell mass) [12,13]. Both chronic fluid overload and malnutrition are well-recognized factors contributing to the high morbidity and mortality in hemodialysis patients. However, there are many confounding interactions between fluid volume and nutritional status in chronic hemodialysis patients, especially in the context of the MIA complex. For example, fluid overload and bowel edema could lead to altered gut permeability to bacteria and/or endotoxins, and the subsequent translocation of these materials into the circulation. Translocated bacteria and/or endotoxins within the circulation could be an important stimulus of systemic immune activation and cytokine production [9]. In chronic hemodialysis patients, fluid overload-induced arterial distension is also associated with increased aortic PWV (Laplace’s law); better management of fluid overload leads to a significant decrease in arterial PWV and stiffness [10, 20]. Conversely, inflammation-induced hypoalbuminemia and increased vascular permeability enhance extravascular fluid shift, thereby resulting in ECF volume overload [11]. It is also well known that inflammatory conditions can cause loss of muscle mass through the activation of the ubiquitin-proteasome proteolytic system [21]. This depletion in body cell mass could eventually lead to a decrease in the ICF volume and a relative increase in the ECF/ICF volume ratio.

In view of the above considerations, the ECF/ICF ratio was postulated to be highly-related to the MIA complex and could be defined as a novel integrated marker reflecting both fluid overload and malnutrition. In this study, the high ECF/ICF ratio group had higher serum CRP, PWV, and plasma BNP, as well as lower serum albumin, nPCR, and SGA score, all of which are MIA and fluid volume components. The ECF/ICF ratio was also found to be well-correlated with all of the MIA and fluid volume components, and nullified the effects of the individual MIA-fluid volume components on all-cause mortality and new CVD in multivariate analysis. As such, the ECF/ICF ratio can be used as an integrated marker reflecting both the MIA complex and fluid volume status in chronic hemodialysis patients.

In this study, a higher ECF/ICF ratio was associated with a significantly higher all-cause mortality and new CVD. The higher ECF/ICF ratio group also had higher systolic BP, pulse pressure, LVMI, and number of ingested anti-hypertensive pills, all of which can also be indirect markers of underlying fluid volume excess and vascular stiffness. Furthermore, the mean age and proportions of patients with diabetes and prior CVD were also higher in the higher ECF/ICF ratio group. These results suggest that in elderly patients with diabetes and prior CVD, specific attention for the possible presence of fluid overload and malnutrition are highly required.

In the unadjusted univariate Cox analysis, many traditional and non-traditional risk factors such as MIA components, fluid volume marker, diabetes, age, systolic BP, and ankle brachial index were significant risk factors for all-cause mortality and new CVD. However, in adjusted multivariate Cox analysis, only the ECF/ICF ratio and prior CVD were significant risk factors for both all-cause mortality and new CVD, which again underlies the importance and clinical utility of the ECF/ICF ratio as an integrative and universal risk indicator in chronic hemodialysis patients.

The strengths of our investigation are its prospective design, consistent definition of the ECF/ICF ratio and MIA-fluid volume components and the complete longitudinal ascertainment of all-cause mortality and new CVD. As a measure for body fluid compartment volume, MF-BIA is highly reproducible (coefficient of variation<1.8); therefore, any biologically-important changes within the ECF and TBF, which can occur in many acute and chronic illnesses, could be detected at the bedside with this technique [14, 22]. Previous body composition studies used the ECF/TBF ratio [23, 24] or the sole ECF volume based overhydration index [8, 20, 25–27] as an indicator of the fluid volume status. However, the ECF/TBF ratio could not accurately reflect the changes within the body fluid volume; this is because a change in the ECF volume is accompanied by a simultaneous change in the ECF component of the TBF. To prevent this unidirectional change in denominator and numerator, we used the ECF/ICF ratio; this is another major strength of our investigation. Our study showed that, contrary to the ECF/ICF ratio, the ECF/TBF ratio was not an independent predictor of all-cause mortality and new CVD in the adjusted multivariate Cox analysis, which supports the superiority of the ECF/ICF ratio as risk indicator as compared with the ECF/TBF ratio. The commonly used sole ECF or TBF volume based overhydration index also have shortcoming of not accurately reflecting ICF volume based nutritional status. Our study showed that the dry weight adjusted sole ECF or TBF volume were not a significant risk factor for all-cause mortality and new CVD. The correlations of these ECF or TBF based volume markers to the MIA component and plasma BNP was also inferior as compared to the ECF/ICF ratio.

Our study nevertheless has several limitations. First, we measured the ECF/ICF ratio and MIA-fluid volume component at enrollment; these were not measured serially. Thus, it may not reflect changes over time or time-averaged exposure, although several recent studies [25–27] have shown that a single measurement of volume status by BIA can predict the long-term survival of hemodialysis patients. We also measured the pre-dialytic value of the ECF/ICF ratio to overcome the acute, uneven effect of hemodialysis ultrafiltration on ECF and ICF volumes. Second, there could be a significant inter-individual variability in the BIA-measured body composition estimate, although MF-BIA has relatively good overall agreement to the gold-standard isotope dilution techniques [28]. We speculate that our segmental BIA measurement (rather than whole-body BIA measurement) and perhaps the use of the ECF/ICF ratio value (rather than absolute ECF or ICF volume) potentially yielded more accurate results with much less inter-individual variability. Third, the levels of plasma BNP would be affected by cardiac function as well as volume status, which could decrease the significance of plasma BNP level as a volume marker. However, in this study, there was no difference in the global left ventricular systolic function as assessed by left ventricular ejection fraction between the high and low ECF/ICF ratio group. This finding implies that higher plasma BNP level, as observed in the high ECF/ICF ratio group, is more likely a result of volume excess. Fourth, this is single-center study that enrolled a relatively small numbers of hemodialysis patients, which may account for the exclusion of diabetes as a significant risk factor for both all-cause mortality and new CVD. However this may be due to the underlying association of diabetes with prior CVD and high ECF/ICF ratio that nullify the independent effect of diabetes on all-cause mortality and new CVD. A large prospective multicenter study will help confirm the causal link between the ECF/ICF ratio and all-cause mortality or CVD.

In conclusion, this study demonstrated that chronic hemodialysis patients with high ECF/ICF ratio are not only fluid overloaded, but are also malnourished and have stiff artery with more inflammation. As a novel integrated marker of fluid volume overload and malnutrition, the ECF/ICF ratio is highly-related to the MIA complex, and can be utilized as a major risk indicator for all-cause mortality and CVD in this population of patients.
